# Effectiveness of Omega-3 Polyunsaturated Fatty Acids in Non-Alcoholic Fatty Liver Disease: A Meta-Analysis of Randomized Controlled Trials

**DOI:** 10.1371/journal.pone.0162368

**Published:** 2016-10-06

**Authors:** Xi-Xi He, Xiao-Li Wu, Ren-Pin Chen, Chao Chen, Xiao-Gang Liu, Bin-Jiao Wu, Zhi-Ming Huang

**Affiliations:** 1 Department of Gastroenterology, The First Affiliated Hospital of Wenzhou Medical University, Wenzhou, Zhejiang Province, China; 2 Department of Gastroenterology, Ningxia People’s Hospital, Yinchuan, Ningxia, China; 3 Department of Acupuncture and Moxibustion, The People’s Hospital of Yueqing, Wenzhou, Zhejiang Province, China; East Tennessee State University, UNITED STATES

## Abstract

**Background:**

Non-alcoholic fatty liver disease (NAFLD) is a clinical syndrome with the main characteristic of diffuse liver cells with fatty changes. The clinical evolution of NAFLD includes simple non-alcoholic fatty liver, non-alcoholic steatohepatitis (NASH), liver fibrosis and cirrhosis, and even hepatocellular carcinoma.

**Methods and Findings:**

We conducted this review to identify the effectiveness of omega-3 polyunsaturated fatty acids (ω-3 PUFA) in NAFLD. We searched PubMed, Cochrane Library and Embase. All randomized controlled trials (RCTs) of ω-3 PUFA treatment for NAFLD were considered. Two reviewers assessed the quality of each study and collected data independently. Disagreements were resolved by discussion among the reviewers and any of the other authors of the paper. We performed a meta-analysis and reported summary estimates of outcomes as inverse variance (IV), fixed or random, with 95% confidence intervals (CIs). We included seven RCTs involving 442 patients (227 for the experimental group and 215 for the control group). All the patients were divided into two groups: one treated with ω-3 PUFA and the other was the control group (generally placebo). The demographics of the ω-3 PUFA and control groups were comparable. Beneficial changes in alanine aminotransferase (ALT) (IV 95% CI: −7.61 [−12.83 to −2.39], *p* = 0.004), total cholesterol (TC) (IV 95% CI: −13.41 [−21.44 to −5.38], *p* = 0.001), triglyceride (TG) (IV 95% CI: −43.96 [−51.21 to −36.71], *p*<0.00001) and high-density lipoprotein cholesterol (HDL-C) (IV 95% CI: 6.97 [2.05 to 11.90], *p* = 0.006) favored ω-3 PUFA treatment. Omega-3 PUFA tended towards a beneficial effect on aspartate aminotransferase (AST) (IV 95% CI: −6.89 [−17.71 to 3.92], *p* = 0.21), γ-glutamyl transferase (GGT) (IV 95% CI: −8.28 [−18.38 to 1.83], *p* = 0.11) and low-density lipoprotein cholesterol (LDL-C) (IV 95% CI: −7.13 [−14.26 to 0.0], *p* = 0.05).

**Conclusions:**

Supplementation with ω-3 PUFA is a practical and effective treatment for NAFLD to decrease ALT, TC and increase HDL-C, especially to decrease TG. Omega-3 PUFA also has a tendency toward a beneficial effect on AST, GGT and LDL-C. More high-quality, large RCTs are needed to validate our findings.

## Introduction

Recently, non-alcoholic fatty liver disease (NAFLD) has become more frequent due to multiple factors including diet (high fat, high cholesterol, high sucrose), lifestyle and genetic background. NAFLD is 3.5 times more common in obese than non-obese patients [1]. The narrow definition of NAFLD is intrahepatic triglycerides comprising >5% of the liver wet weight, or fatty changes in >30% of the liver parenchymal cells. In recent years, as our understanding of the disease has increased, it is believed that NAFLD is a genetic, environmental and metabolic liver disease, which leads to energy imbalance. It is a metabolic syndrome in the liver, which is closely related to insulin resistance (IR) and inflammatory cytokines. In a sample of 139 NAFLD patients, metabolic syndrome was found in 25.9% of the sample [2]. Kumar et al. [3] found in a trial of 79 patients that there was a significant increase in proinflammatory cytokines and a significant association of interleukin (IL)-6 with IR in NAFLD. NAFLD can be an independent disease, or a pathological process of a systemic disease in the liver. Non-alcoholic steatohepatitis (NASH) is a more severe form of NAFLD, which can develop into liver cirrhosis and end-stage liver disease through liver fibrosis.

Polyunsaturated fatty acids (PUFA) are necessary in humans and often divided into ω-3 PUFA and ω-6 PUFA. A study [4] has shown that ω-6 PUFA are mainly composed of linoleic acid (LA), and ω-3 PUFA are mainly composed of α-linolenic acid (ALA). LA is metabolized to arachidonic acid, and ALA is metabolized to eicosapentaenoic acid (EPA) and docosahexaenoic acid (DHA). The balance between ω-3 and ω-6 PUFA is important in human health [5], and the optimum ratio in the diet is 4 to 1 [6]. However, we have found that most NAFLD patients have a higher level of ω-6 and a lower level of ω-3 PUFA [4–7], and the ratio can be 20–25: 1 [5]. Thus, supplementation with ω-3 PUFA is necessary, especially for the prevention and treatment of NAFLD.

Therefore, we conducted a meta-analysis of RCTs on ω-3 PUFA treatment for NAFLD to determine the effects of ω-3 PUFA supplementation on blood markers of liver injury and dyslipidemia.

## Methods

### Search Strategy

We adhered to the Preferred Items for Systematic Reviews and Meta-Analyses (PRISMA) guidelines (S1 File). We used a multi-method iterative approach to identify relevant studies in Medline PubMed, Cochrane Library and Embase using the following search terms: ‘fish oil’, ‘EPA’ (eicosapentaenoic acid), ‘DHA’ (docosahexaenoic acid), ‘omega-3 PUFA’ (ω-3 PUFA), ‘n-3 PUFA’, and ‘NAFLD’, ‘fatty liver’, ‘hepatic steatosis’, ‘hepatic liver’, ‘steatohepatitis’, and ‘NASH’. There were no restrictions on publication language. The detailed search strategies for Medline PubMed, Cochrane Library and Embase have been provided in S2 File.

### Study Selection and Data Extraction

According to the PICO (population, intervention, comparison, and outcomes) principle, the criteria for study inclusion were: (i) patients with NAFLD or NASH; (ii) intervention with ω-3 PUFA; (iii) comparison of intervention: control group (mostly placebo); (iv) outcome: primary results, changes in alanine aminotransferase (ALT), aspartate aminotransferase (AST), γ-glutamyl transferase (GGT), total cholesterol (TC), triglyceride (TG), low-density lipoprotein cholesterol (LDL-C) and high-density lipoprotein cholesterol (HDL-C), and secondary results, changes in fasting glucose, homeostatic model assessment insulin resistance (HOMA_IR_), adiponectin, liver fat and fibrosis; and (v) study design: randomized control trials (RCTs). In the search process, we excluded the following articles: clinical trials involving children; two studies on adults were excluded because of study design and rationale of the preconceived trial [8, 9]; one studied NAFLD combined with diabetes [10]; one was published only as a conference abstract [11]; and two were excluded because we could not calculate the data of changes (mean±SD) according to the data they gave [12, 13]. Article selection was conducted independently by two of the authors. All disagreements were resolved by discussion among these two authors and any of the other authors of the paper.

### Methodological Quality Evaluation

The methodological quality assessment was based on modified JADAD score [14], including the following: sequence generation, allocation concealment, blinding, withdrawals and dropouts, and randomization efficacy. The evaluation process was independently performed by two of the authors. Disagreement was resolved by consensus after discussion.

### Statistical Analysis

Meta-analysis was performed using the Cochrane Collaboration’s Review Manager Software RevMan 5.3. The outcomes are presented with 95% confidence intervals (CIs). The changes (mean±SD) in ALT, AST, GGT, TG, TC, HDL-C and LDL-C were extracted after ω-3 PUFA and control treatment. We sent e-mails to seven authors to ask for their original data of the change (mean±SD) in the two groups (the ω-3 PUFA group and the control group), but only one replied [15]. As a result, we calculated the mean±SD in the other six reviews according to the Cochrane Handbook for Systematic Reviews of Interventions. The mean (change) was mean (basic)—mean (end), and we calculated the R (constant) using the formula of “SD (change) × SD (change) = SD (basic) × SD (basic) + SD (end) × SD (end) −2 × R (constant) × SD (basic) × SD (end)” in ALT, AST, GGT, TC, TG, HDL-C and LDL-C according to the original data which were provided by the author Sofi et al. [15], and then we added R (constant) into this formula to calculate the SD (change) in the other six articles. A random-effects or fixed-effects model was used in the presence and absence of statistical heterogeneity, respectively. We also analyzed fasting glucose, HOMA_IR_, adiponectin, liver fat and fibrosis in these RCTs.

## Results

**Fig 1:**
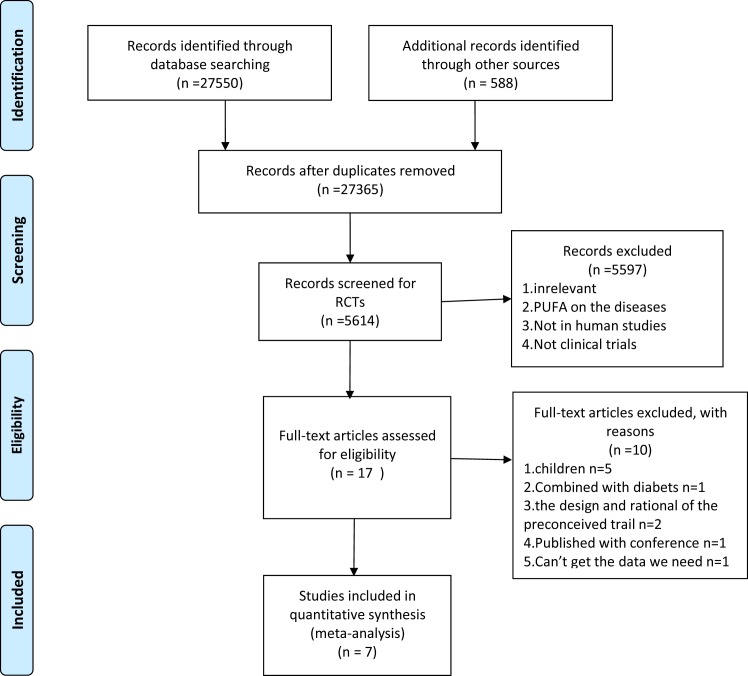
Flow diagram representing the selection process.

We included seven RCTs [15–21] in our analysis. A flow diagram of the study selection process is shown in Fig 1. The characteristics of the included studies are summarized in Table 1, and the quality of the included RCTs are shown in Table 2. Baseline demographic data of the subjects in these RCTs are shown in Table 3. We analyzed ALT, AST, GGT, TC, TG, LDL-C and HDL-C, and presented the results as forest plots. We established the subgroups according to the treatment time (6 months and ≥12 months) and the dose of the ω-3 PUFA (<3 g and ≥3 g). We also analyzed fasting glucose, HOMA_IR_, adiponectin, liver fat and fibrosis in these trials.

**Table 1:** Characteristics of included RCTs.

	Total no.	Disease	Dose of ω-3 PUFA	Duration	Control
Purrello, et al.(2007) [16]	36	NAFLD	2g	6mon	AHA
Feng-Shang Zhu, et al.(2008) [17]	134	NAFLD	2g	6mon	Placebo
Qing Xie, et al.(2008) [18]	46	NAFLD	4–5g	6mon	Placebo
Sofi, et al.(2010) [15]	11	NAFLD	0.83g	12mon	Placebo
Byrne, et al.(2014) [19]	103	NAFLD	4g	15–18mon	Placebo
Stephen, et al.(2015) [21]	34	NASH	3g	12mon	Placebo
Lu-Hua Yang, et al.(2015) [20]	78	NASH	6.4g	6mon	Normal saline

NAFLD, non-alcoholic fatty liver disease; AHA, alpha hydrxy acid; NASH, non-alcoholic steatohepatitis

**Table 2:** Quality of included RCTs.

	Randomization method	Blind method	Jadad score
Purrello, et al.(2007) [16]	Random sampling	None	6
Feng-Shang Zhu, et al.(2008) [17]	Not stated	None	4
Qing Xie, et al.(2008) [18]	Not stated	Double blind	4
Sofi, et al.(2010) [15]	Not stated	None	6
Byrne, et al.(2014) [19]	Not stated	None	5
Stephen, et al.(2015) [21]	Stratified block 1:1 randomization scheme	Double blind	6
Lu-Hua Yang, et al.(2015) [20]	Not stated	Double blind	4

**Table 3:** Baseline demographic data of the subjects in the RCTs.

Parameters	Control(n = 215)	ω-3 PUFA(n = 227)	*p value*
Median age (y)	49.4	49.2	>0.05
Gender			
Male	147	151	>0.05
Female	68	76	>0.05

### Primary results

#### ALT

**Fig 2:**
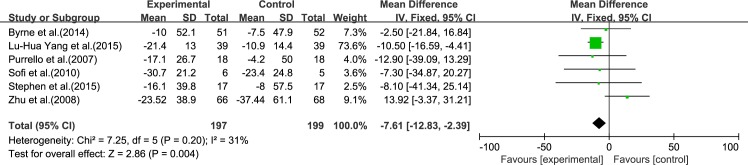
Forest plot in analysis of ω-3 PUFA for NAFLD on ALT.

Six reviews [15–17, 19–21] reported results for ALT. Using a fixed-effect model, we showed that ω-3 PUFA therapy had a significant effect on ALT [inverse variance (IV): −7.61, 95% CI: −12.83 to −2.39], *p* = 0.004). There was no significant heterogeneity among the studies (*I*^2^ = 31%, p = 0.20), (Fig 2).

#### AST

**Fig 3:**
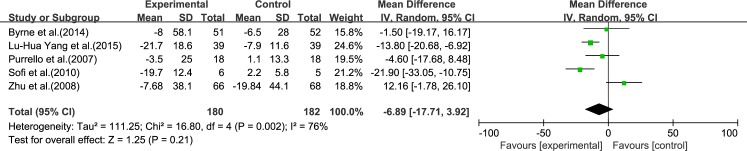
Forest plot in analysis of ω-3 PUFA for NAFLD on AST.

Five reviews [15–17, 19, 20] reported results for AST. Significant heterogeneity was found among the studies (*I*^2^ = 76%, *p* = 0.002). Using a random-effect model, we showed that ω-3 PUFA therapy had an effect on AST, but this did not reach statistical significance (IV −6.89, 95% CI: −17.71 to 3.92, *p* = 0.21), (Fig 3).

#### GGT

**Fig 4:**
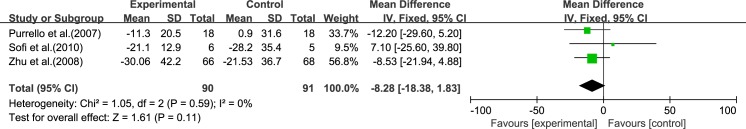
Forest plot in analysis of ω-3 PUFA for NAFLD on GGT.

Three reviews[15–17] reported results for GGT. Using a fixed-effect model, we showed that ω-3 PUFA therapy had an effect on GGT, but this did not reach statistical significance (IV: −8.28, 95% CI: −18.38 to 1.83, *p* = 0.11). There was no significant heterogeneity among the studies (*I*^2^ = 0%, *p* = 0.59), (Fig 4).

#### TC

**Fig 5:**
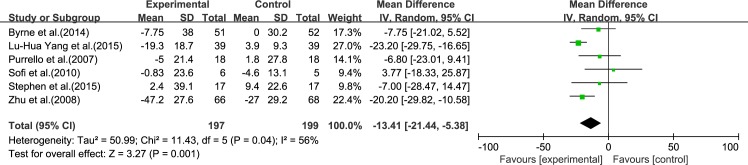
Forest plot in analysis of ω-3 PUFA for NAFLD on TC.

Six reviews [15–17, 19–21] reported results for TC. Significant heterogeneity was found among the studies (*I*^2^ = 56%, *p* = 0.04). Using a random-effect model, we showed that ω-3 PUFA therapy had a significant effect on TC (IV: −13.41, 95% CI: −21.44 to −5.38, *p* = 0.001), (Fig 5).

#### TG

**Fig 6:**
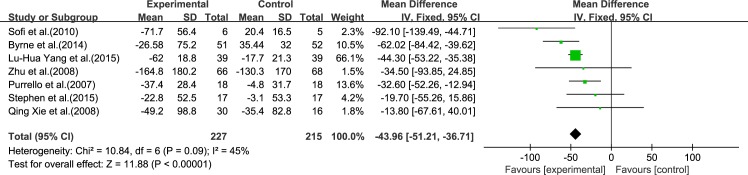
Forest plot in analysis of ω-3 PUFA for NAFLD on TG.

Seven reviews [15–21] reported results for TG. Using a fixed-effect model, we showed a significant effect of ω-3 PUFA therapy on TG (IV: −43.96, 95% CI: −51.21 to −36.71, *p*<0.00001). There was no significant heterogeneity among the studies (*I*^2^ = 45%, *p* = 0.09), (Fig 6).

#### LDL-C

**Fig 7:**
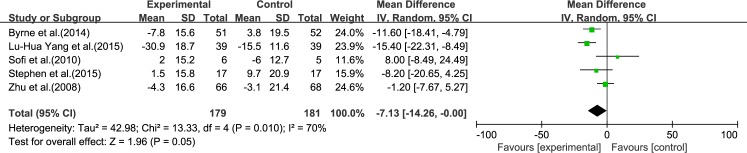
Forest plot in analysis of ω-3 PUFA for NAFLD on LDL-C.

Five reviews [15, 17, 19–21] reported results for LDL-C. Significant heterogeneity was found among the studies (*I*^2^ = 70%, *p* = 0.010). Using a random-effect model, we showed that ω-3 PUFA therapy had an effect on LDL-C, but this did not reach statistical significance (IV: −7.13, 95% CI: −14.26 to −0.0, *p* = 0.05), (Fig 7).

#### HDL-C

**Fig 8:**
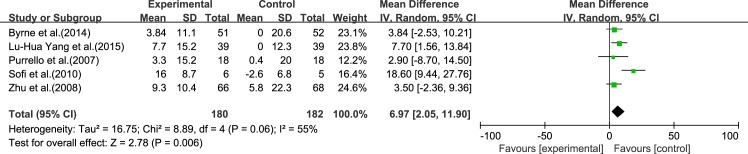
Forest plot in analysis of ω-3 PUFA for NAFLD on HDL-C.

Five reviews [15–17, 19, 20] reported results for HDL-C. Significant heterogeneity was found among the studies (*I*^2^ = 55%, *p* = 0.06). Using a random-effect model, we showed that ω-3 PUFA therapy had a significant effect on HDL-C (IV: 6.97, 95% CI: 2.05–11.90, *p* = 0.006), (Fig 8).

#### Heterogeneity

We have found some heterogeneity in our analysis of some parameters, including AST, TC, HDL-C and LDL-C. We used the method of “exclusion of one trial each time” in heterogeneity analysis. The *I*^2^ and P results are summarized in Table 4. We also performed a sensitivity analysis to detect heterogeneity for AST, TC, HDL-C and LDL-C according to the JADAD score, follow-up time, dose, BMI and proportion of male patients (Tables 5 and 6). We found that when the trials by Zhu et al. [17], Yang et al.[20] and Sofi et al.[15] were excluded, the heterogeneity decreased. We also found that, if the proportion of male patients was <60% or BMI was >30, the heterogeneity was reduced to 0%.

**Table 4:** Analysis of heterogeneity by “exclusion of one trial each time” method.

	Authors	I^2^ ^a^	P ^b^	IV 95% CI ^c^
AST;I^2^ = 76%, *p* = 0.21	Byrne, et al.(2014)	81%	0.22	-7.81[-20.30,4.67]
	Lu-Hua Yang, et al.(2015)	79%	0.57	-4.41[-19.53,10.71]
	Purrello, et al.(2007)	81%	0.29	-7.22[-20.68,6.25]
	Sofi, et al.(2010)	74%	0.64	-2.88[-14.89,9.12]
	Feng-Shang Zhu, et al.(2008)	47%	0.002	-12.13[-19.98,-4.27]
TC;I^2^ = 56%, *p* = 0.001	Byrne, et al.(2014)	56%	0.001	-14.60[-23.43,-5.76]
	Lu-Hua Yang, et al.(2015)	30%	0.01	-10.48[-18.63,-2.34]
	Purrello, et al.(2007)	58%	0.001	-14.46[-23.18,-5.74]
	Sofi, et al.(2010)	48%	<0.001	-15.78[-23.17,-8.39]
	Stephen, et al.(2015)	62%	0.002	-13.96[-22.67,-5.25]
	Feng-Shang Zhu, et al.(2008)	64%	0.05	-10.52[-21.24,0.20]
LDL-C;I^2^ = 70%, *p* = 0.05	Byrne, et al.(2014)	75%	0.26	-5.43[-14.93,4.06]
	Lu-Hua Yang, et al.(2015)	60%	0.22	-4.68[-12.17,2.81]
	Sofi, et al.(2010)	68%	0.009	-9.12[-15.98,-2.27]
	Stephen, et al.(2015)	77%	0.12	-6.73[-15.26,1.80]
	Feng-Shang Zhu, et al.(2008)	56%	0.01	-9.48[-16.76,-2.21]
HDL-C;I^2^ = 55%, *p* = 0.06	Byrne, et al.(2014)	63%	0.01	8.00[1.66,14.35]
	Lu-Hua Yang, et al.(2015)	65%	0.04	6.91[0.24,13.58]
	Purrello, et al.(2007)	65%	0.008	7.64[1.98,13.30]
	Sofi, et al.(2010)	0%	0.005	4.82[1.44,8.19]
	Feng-Shang Zhu, et al.(2008)	60%	0.01	8.16[1.89,14.43]

^a^ *I^2^*

^b^ *P*

^c^ *IV 95% CI* were calculated using Cochrane Collaboration’s Review Manager Software RevMan 5.3.

**Table 5:** Sensitivity analysis of heterogeneity on AST and TC.

	AST				TC			
Strata strategy	References	I^2^ ^a^	P ^b^	IV 95% CI ^c^	References	I^2^ ^d^	P ^e^	IV 95% CI ^f^
**Based on JADAD score**								
High quality RCTs (>4 score)	15,16,19	64%	0.13	-10.36[-23.64,2.91]	15,16,19,21	0%	0.20	-5.65[-14.1,2.90]
Low quality RCTs (≤4 score)	17,21	91%	0.90	-1.56[-26.96,23.84]	17,20	0%	<0.001	-22.25[-27.66,-16.83]
**Based on follow-up time**								
Long-term (≥12mon)	15,19	73%	0.20	-12.90[-32.75,6.95]	15,19,21	0%	0.31	-5.20[-15.26,4.85]
Short-term (6mon)	16,17,21	82%	0.70	-2.98[-18.08,12.12]	16,17,20	41%	<0.01	-19.45[-26.88,-12.01]
**Based on dose**								
Higher dose(≥3g)	19,20	38%	0.06	-10.45[-21.18,0.28]	19,20,21	64%	0.02	-14.80[-27.10,-2.50]
Lower dose(<3g)	15,16,17	86%	0.61	-5.13[-24.67,14.41]	15,16,17	59%	0.14	-10.35[-24.15,3.45]
**Based on BMI**								
Higher BMI(>30)	16,19	0%	0.51	-3.50[-14.02,7.01]	16,19,21	0%	0.12	-7.30[-16.56,1.96]
Lower BMI(<30)	15,17,20	87%	0.30	-8.58[-24.96,7.80]	15,17,20	62%	0.0007	-17.84[-28.10,-7.58]
**Based on proportion of male patients**								
Higher male ratio (>60%)	15,17,20	87%	0.30	-8.58[-24.96,7.80]	15,17,20	62%	0.0007	-17.84[-28.10,-7.58]
Lower male ratio (<60%)	16,19	0%	0.51	-3.50[-14.02,7.01]	16,19,21	0%	0.12	-7.30[-16.56,1.96]

BMI, Body Mass Index; AST, aspartate aminotransferase; TC, total cholesterol

^a^ *I^2^*

^b^ *P*

^c^ *IV 95% CI*

^*d*^ *I^2^*

^e^ *P*

^f^ *IV 95% CI* were calculated by Cochrane Collaboration’s Review Manager Software RevMan 5.3.

**Table 6:** Sensitivity analysis of heterogeneity on HDL-C and LDL-C.

	HDL-C				LDL-C			
Strata strategy	References	I^2^ ^a^	P ^b^	IV 95% CI ^c^	References	I^2^ ^d^	P ^e^	IV 95% CI ^f^
**Based on JADAD score**								
High quality RCTs (>4 score)	15,16,19	73%	0.10	8.45[-1.47,18.38]	15,19,21	57%	0.24	-6.08[-16.15,4.00]
Low quality RCTs (≤4 score)	17,21	50%	0.01	5.50[1.27,9.74]	17,20	88%	0.25	-8.25[-22.16,5.67]
**Based on follow-up time**								
Long-term (≥12mon)	15,19	85%	0.14	10.84[-3.61,25.28]	15,19,21	57%	0.24	-6.08[-16.15,4.00]
Short-term (6mon)	16,17,21	0%	0.01	5.20[1.11,9.18]	17,20	88%	0.25	-8.25[-22.16,5.67]
**Based on dose**								
Higher dose(≥3g)	19,20	0%	0.01	5.84[1.42,10.26]	19,20,21	0%	<0.001	-12.78[-17.30,-8.26]
Lower dose(<3g)	15,16,17	65%	0.04	6.91[0.24,13.58]	15,17	4%	0.96	0.15[-6.23,6.52]
**Based on BMI**								
Higher BMI(>30)	16,19	0%	0.20	3.62[-1.96,9.21]	19,21	0%	0.004	-10.82[-16.79,-4.84]
Lower BMI(<30)	15,17,20	73%	0.02	9.21[1.54,16.88]	15,17,20	83%	0.50	-4.30[-16.71,-8.10]
**Based on proportion of male patients**								
Higher male ratio (>60%)	15,17,20	73%	0.02	9.21[1.54,16.88]	15,17,20	83%	0.50	-4.30[-16.71,-8.10]
Lower male ratio (<60%)	16,19	0%	0.20	3.62[-1.96,9.21]	19,21	0%	0.004	-10.82[-16.79,-4.84]

BMI, Body Mass Index; HDL-C, high-density lipoprotein cholesterol; LDL-C:low-density lipoprotein cholesterol

^a^ *I^2^*

^b^ *P*

^c^ *IV 95% CI*

^*d*^ *I^2^*

^e^ *P*

^f^ *IV 95% CI* were calculated using Cochrane Collaboration’s Review Manager Software RevMan 5.3.

### Subgroup Analysis

#### Treatment time

**Fig 9:**
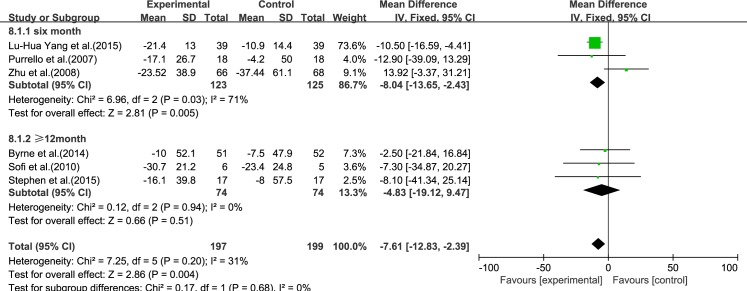
Forest plot in subgroup analysis of the treatment time on ALT.

**Fig 10:**
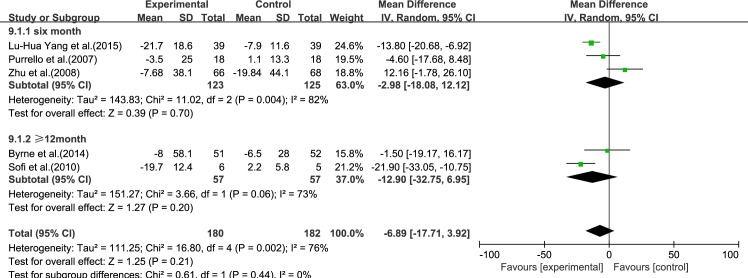
Forest plot in subgroup analysis of the treatment time on AST.

**Fig 11:**
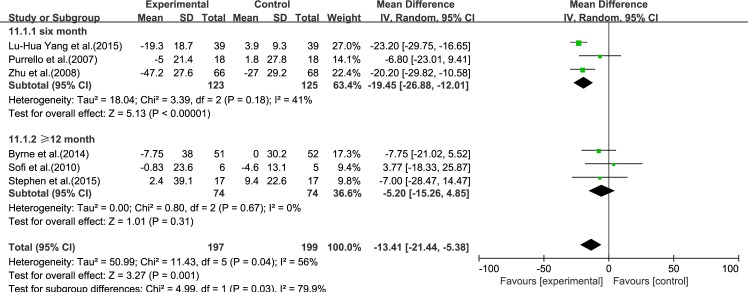
Forest plot in subgroup analysis of the treatment time on TC.

**Fig 12:**
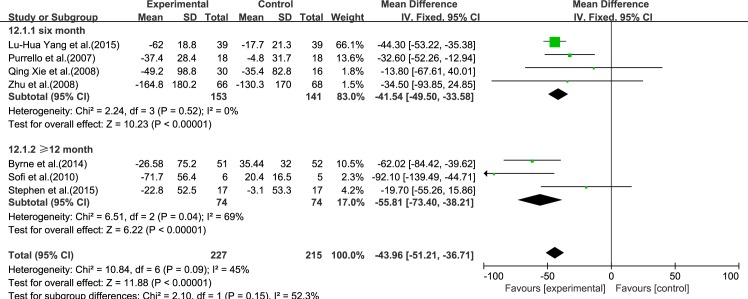
Forest plot in subgroup analysis of the treatment time on TG.

**Fig 13:**
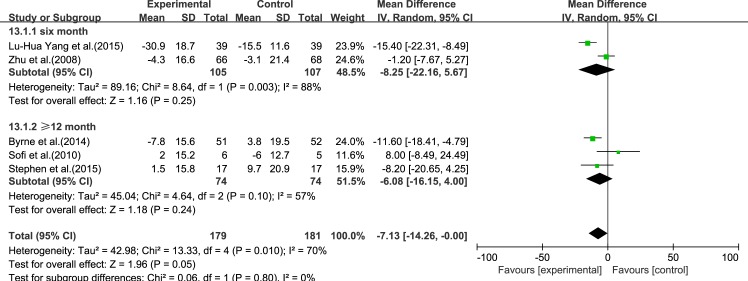
Forest plot in subgroup analysis of the treatment time on LDL-C.

**Fig 14:**
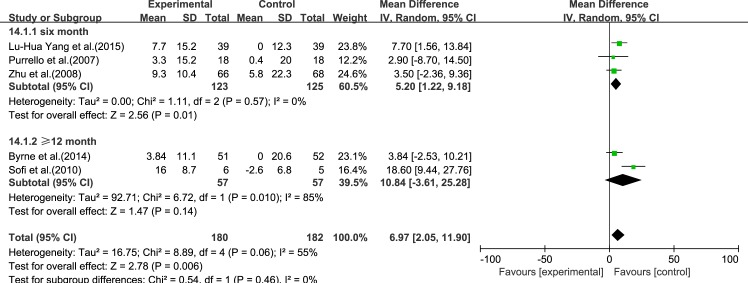
Forest plot in subgroup analysis of the treatment time on HDL-C.

We analyzed the reviews according to treatment time of 6 months and ≥12 months, except for GGT, for which there were only three trials. For ALT, LDL-C and TC, the effectiveness of 6 months treatment was better than ≥12 months, without considering the heterogeneity. For AST, HDL-C and TG, the effectiveness of ≥12 months treatment was better than 6 months, without considering the heterogeneity (Figs 9–14).

#### Dose

**Fig 15:**
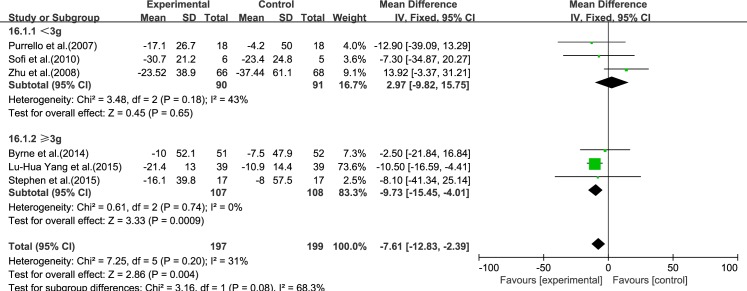
Forest plot in subgroup analysis of the dose of ω-3 PUFA on ALT.

**Fig 16:**
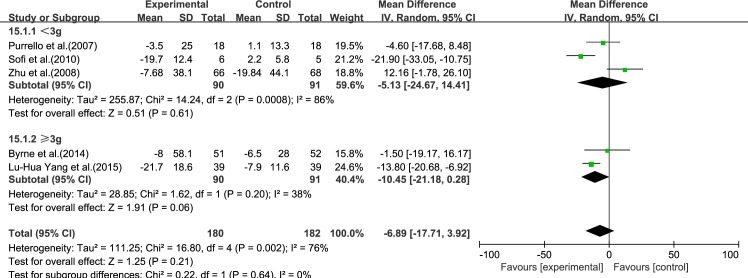
Forest plot in subgroup analysis of the dose of ω-3 PUFA on AST.

**Fig 17:**
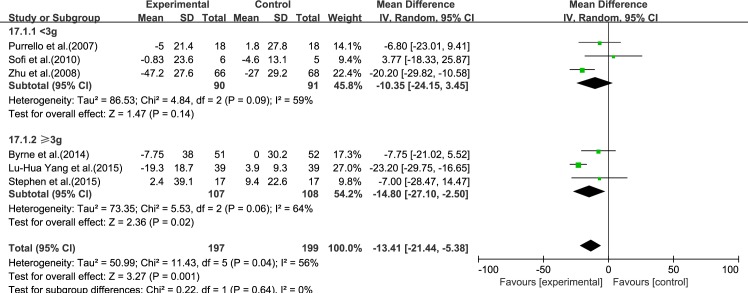
Forest plot in subgroup analysis of the dose of ω-3 PUFA on TC.

**Fig 18:**
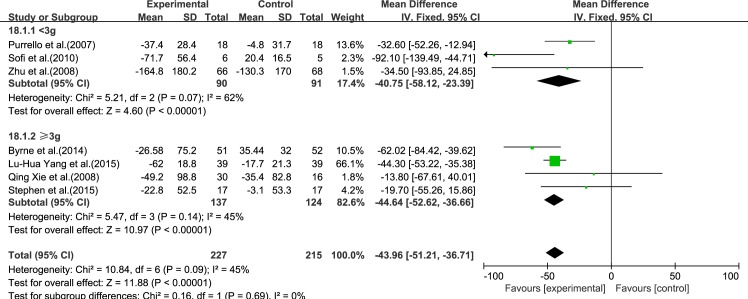
Forest plot in subgroup analysis of the dose of ω-3 PUFA on TG.

**Fig 19:**
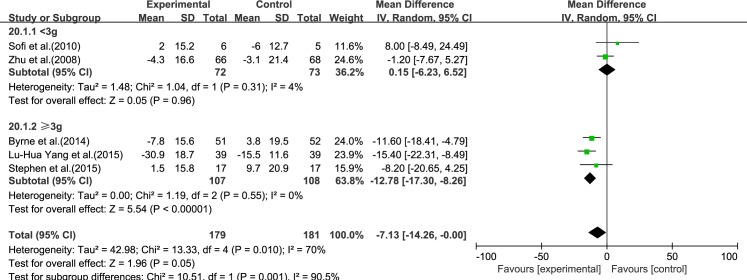
Forest plot in subgroup analysis of the dose of ω-3 PUFA on LDL-C.

**Fig 20:**
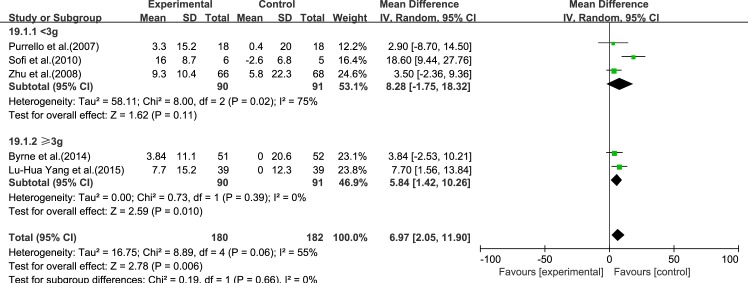
Forest plot in subgroup analysis of the dose of ω-3 PUFA on HDL-C.

We analyzed the reviews according to the dose of ω-3 PUFA (<3 and ≥3 g), except for GGT, for which the dose was the same in each trial. For ALT, AST, LDL-C, TC and TG, the effectiveness of ≥3 g treatment was better than <3 g, without considering the heterogeneity. Only for HDL-C was the effectiveness of <3 g treatment better than ≥3 g, without considering the heterogeneity (Figs 15–20).

### Secondary Results

#### Fasting glucose

Four trials mentioned fasting glucose in their results [15, 19–21]. Only the trial by Byrne et al. [19] showed that serum fasting glucose level increased in the control group and decreased in the ω-3 PUFA group.

#### HOMA_IR_

IR was reported in three trials [15, 16, 21]. In the trials of Purrello et al. [16] and Sofi et al. [15], HOMA_IR_ decreased in both the control and the ω-3 PUFA groups, with a more significant decrease in the ω-3 PUFA group.

#### Adiponectin

Adiponectin increased in both the control and ω-3 PUFA groups in two trials [15, 21], with a greater increase in the ω-3 PUFA group (*p* = 0.04) [15].

#### Liver fat

Three trials mentioned the level of liver fat in their results [17, 19, 21]. In the trials of Stephen et al. [21] and Byrne et al. [19], there was a greater decrease in liver fat in the ω-3 PUFA group compared to the control group.

#### Fibrosis

Three trials mentioned fibrosis in their results [19–21], and only the trial by Lu-Hua Yang et al. [20] showed that ω-3 PUFA therapy was beneficial for improving hepatic fibrosis by reducing serum levels of type Ⅳ collagen and P-Ⅲ-P (pro-collagen type III pro-peptide).

### Publication Bias

**Fig 21:**
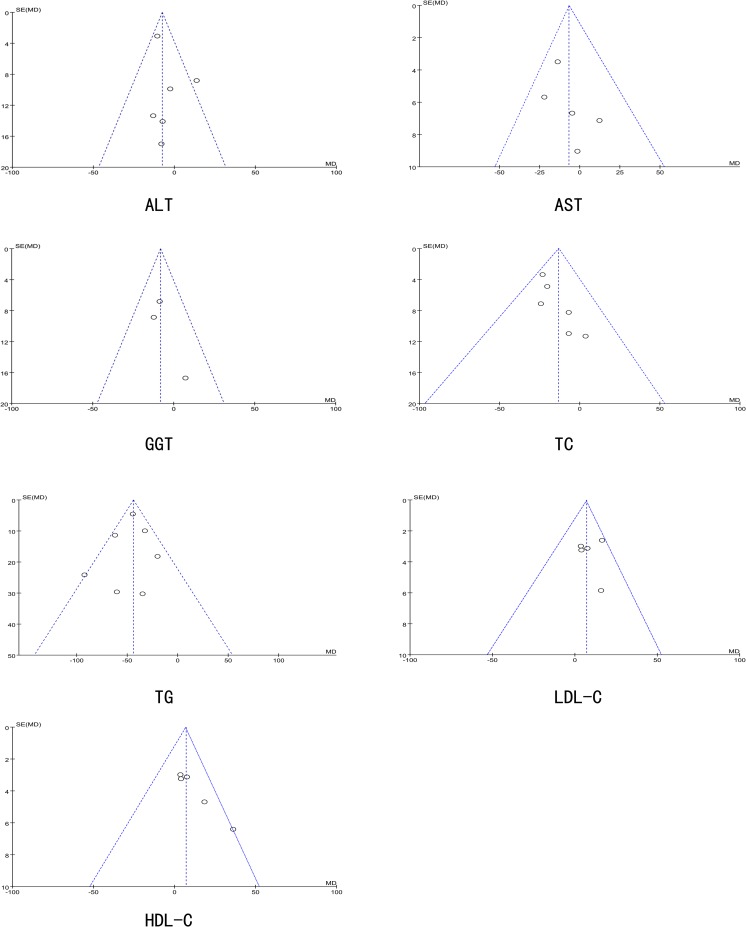
The Funnel plot of Publication Bias.

The funnel plots of ALT, AST, GGT, TC, TG, LDL-C and HDL-C are shown in Fig 21. There was no evidence of publication bias in the present study, and each plot displayed an approximately symmetrical funnel shape

## Discussion

This analysis was a systematic review of RCTs investigating the efficacy of ω-3 PUFA in the treatment of NAFLD. Seven RCTs involving 227 patients in the experimental groups and 215 in the control groups were included in the analysis. Our results support the use of ω-3 PUFA supplementation as a practical and effective treatment for NAFLD, especially for reducing triglycerides. This conclusion is the same as that reached by Parker et al. [22] who first reported ω-3 PUFA supplementation in humans with NAFLD. In the analysis by Parker et al., ω-3 PUFA therapy had a significant beneficial effect on AST and a tendency toward a beneficial effect on ALT, but these effects were not significant after analysis of only RCT data. In this review, ω-3 PUFA therapy had a significant effect on liver fat, whether only RCT data (ES: −0.96, 95% CI: −0.43 to −1.48; *p*<0.001) or all the trials (ES: −0.97, 95% CI: −0.58 to −1.35; *p*<0.001) were included. In a previous meta-analysis (2010) [23], ω-3 PUFA also improved biochemical and radiological markers of NAFLD in a small subgroup of RCTs. Our review has clinical value as the results were extracted from RCTs. We also analyzed more markers, including primary results for ALT, AST, GGT, TC, TG, LDL-C and HDL-C and secondary results including fasting glucose, HOMA_IR_, adiponectin, liver fat and fibrosis. We also analyzed subgroups according to the treatment time and the dose of ω-3 PUFA therapy.

Heterogeneity was identified in the results for AST, TC, HDL-C and LDL-C. We compared subgroups to analyze the heterogeneity. We found that ω-3 PUFA treatment time was insignificant in the heterogeneity analysis, and we could not get the conclusion that the longer the administration time of ω-3 PUFA the better the effect on NAFLD. With regard to the dose of ω-3 PUFA, the effectiveness of ≥3 g treatment was better than <3 g, except for HDL-C. Future investigations should include more RCTs with different doses and treatment times for NAFLD, and this may result in a guide for clinical treatment. When we used the method of “exclusion of one trial each time” and performed a sensitivity analysis of heterogeneity according to the JADAD score, follow-up time, dose, BMI and proportion of male patients to detect heterogeneity, we obtained the same results. Higher BMI (BMI>30) and lower proportion of male patients (<60%) were just in the reference when we excluded the studies by Zhu et al. [17], Yang et al. [20] and Sofi et al. [15], therefore we considered that the heterogeneity may have been due to the sex ratio and BMI. From our data analysis, we found that supplementation with ω-3 PUFA is a practical and effective treatment for NAFLD, especially for reducing triglycerides, and individuals with a high BMI combined with hypertriglyceridemia may provide information as to why ω-3 PUFA reduce triglycerides to a higher degree. However, there have been no RCTs or clinical studies on the association between BMI and the effects of ω-3 PUFA, and this may warrant further investigation. With regard to the lower proportion of male patients, it was recently reported that women with NAFLD are more sensitive to ω-3 PUFA than men with NAFLD [24], however, the reasons for this difference require further study. It should be noted that in the trial by Lu-Hua Yang et al. [20], the dose of ω-3 PUFA was much higher than that used in other trials, and in the trial by Sofi et al. [15], there were only 11 patients in two groups, which may have caused heterogeneity.

In the secondary results, we found that the efficacy of ω-3 PUFA in decreasing fasting glucose, IR, liver fat, fibrosis and increasing adiponectin in our trials was unclear, although an RCT [25] found that ω-3 PUFA improved insulin sensitivity. Many studies have reported an association between IR and NAFLD [26–28]. We also found that adiponectin is an insulin-sensitizing hormone, which can improve IR in rats [29]. Adiponectin can predict the development of type 2 diabetes and coronary heart disease. In clinical trials, adiponectin was found to be involved in resistance to diabetes, arterial atherosclerosis and inflammation, thus the significant increase in adiponectin in our trial [15] was encouraging (*p* = 0.04). As NAFLD is a fatty change in the liver, ω-3 PUFA may be considered an effective method for decreasing liver fat, however, the improvement in liver fat percentage was not very significant in our trials, and it may take a long time to reduce liver fat following treatment with ω-3 PUFA. The conclusion of the trial [20] that ω-3 PUFA therapy was beneficial for improving fibrosis by reducing type IV collagen and P-III-P was encouraging, however, in a mouse model of NASH [30], ω-3 PUFA worsened fibrosis. Thus, the effect of ω-3 PUFA supplementation on liver fibrosis requires further research in the future.

Two RCTs were not included in the analysis due to data format issues. The trial by Arun [12] was rejected as it only provided the median (the 25th and 75th percentiles), and we were unable to calculate the mean changes from these data. However, this trial also showed that EPA reduced the levels of TG (an decrease of 6.5 mg/dL in the EPA group vs. an increase of 12 mg/dL in the placebo group; *p* = 0.03), but it also showed no histological effects on NASH. In addition, there were no significant changes in the fibrosis scores in the treatment group. The RCT by Waitzberg [13] was also rejected as the basic and end data were shown as 95% CI and we were unable to calculate the SD of the changes, and the results showed the number of stabilized patients with improvement after the intervention. However, the trial also reported a significant reduction in serum TG levels compared to baseline (*p* = 0.01) after 3 months of treatment in the ω-3 PUFA group, which was not seen in the placebo group. Serum fasting glucose level increased in the control group and decreased in the ω-3 PUFA group. However, no significant changes in liver steatosis or fibrosis in both the ω-3 PUFA and placebo groups were observed.

There are currently no definitive treatments for NAFLD. Some small trials have shown unconfirmed treatment outcomes. In a study of 56 participants [31], lifestyle modification, including low-fat diet and moderate exercise, improved liver histology after 6 months of this intervention, which emphasizes the importance of lifestyle in NAFLD. Another study [32] showed that losing at least 7% of body weight and reducing caloric intake could be beneficial in patients with NAFLD. Pioglitazone has an obvious effect in NASH [33], both on metabolic and histological parameters [34]. A clinical trial which included 27 patients [35] showed that treatment of NAFLD with rosiglitazone is safe, and leads to significant improvement in liver function and insulin sensitivity. A study [36] of 20 patients with NASH showed that 500 mg metformin three times daily for 4 months significantly improved insulin sensitivity and decreased liver volume by 20%. A recent study [37] also showed that vitamin D supplementation decreased HOMA_IR_ and fasting blood glucose in NAFLD patients. A trial [38] which included 45 patients showed significant improvements in the fibrosis score (*p* = 0.002) after 6 months of vitamin treatment, without significant side effects. A pilot study of 23 patients with NASH [39] also found that vitamins C and E improved serum ALT, thioredoxin, and high-sensitivity C-reactive protein levels and liver histology. Changes in diet can cause an increasing number of metabolic diseases, including NAFLD. A systematic review by White et al. [40] supported an association between NAFLD or NASH and an increased risk of hepatocellular carcinoma. A study from 2004 to 2009 [41] also found that NAFLD was a major cause of hepatocellular carcinoma in the USA, and NAFLD increased the risk of 1-year mortality (odds ratio 1.21; 95% CI: 1.01–1.45). Thus, the treatment of NAFLD has become increasingly important. Recent studies have also shown that supplementation with ω-3 PUFA is also effective in children with NAFLD. Three RCTs [42–44] showed that supplementation with ω-3 PUFA improved liver steatosis in children with NAFLD, but not liver fibrosis. However, the latest RCT indicated that ω-3 PUFA did not affect liver steatosis on ultrasound, but improved AST and GGT levels in children with NAFLD [45]. As supplementation with ω-3 PUFA reduces the risk of NAFLD, we also found that ω-3 PUFA has many beneficial effects in NAFLD patients with cardiovascular diseases. In a study with a 10-year follow-up [46], the incidence of cardiovascular diseases was 19% (17/91 patients) in NAFLD patients and 10% (18/182) in controls, with a significantly higher estimated cumulative risk in NAFLD patients than in controls. An updated clinical review [47] also supported a strong association between NAFLD and cardiovascular diseases. In a clinical trial which included 11,323 patients [48], long-term ω-3 PUFA treatment with 1 g daily, had a beneficial effect on mortality from coronary heart disease, myocardial infarction and stroke.

We observed a significant advantage of ω-3 PUFA in reducing ALT, TC and TG and increasing HDL-C in NAFLD/NASH. However, its effectiveness in improving liver fibrosis is unclear. It should be noted that a recent study [49] in mice showed that EPA had a greater triacylglycerol (TG)-reducing effect than DHA, and that DHA had a greater effect than EPA in improving hepatic inflammation, but no difference in fibrosis was observed. Another study [50] found that DHA was superior to EPA in terms of hepatic metabolism and fibrosis. The efficacy of DHA compared with EPA is controversial, and more RCTs are required to determine which is more efficacious. Many non-RCTs have also reported favorable results for ω-3 PUFA in NAFLD and NASH patients [51, 52]. The first controlled trial of ω-3 PUFA supplementation in patients with NAFLD [53] showed that TG decreased in the ω-3 PUFA group, which was consistent with the results of our meta-analysis. However, supplementation with ω-3 PUFA was controversial. It must be noted that in a recent study, ω-3 PUFA provided no benefit over placebo in NASH patients with diabetes [10], and was even inferior to placebo. In addition, ω-3 PUFA worsened inflammation and fibrosis in mice with experimental NASH [54]. A recent study also found that supplementation with 5 g/day fish oil for 6 weeks did not lower inflammatory biomarkers [55]. Another study also demonstrated that dietary EPA+DHA did not improve insulin secretion or skeletal muscle mitochondrial function in non-diabetic patients with IR [56]. Therefore, more studies are required to establish the effectiveness of ω-3 PUFA in NAFLD.

The limitation in this analysis was the data extraction, which included a small quantity of RCTs. The data were calculated using the Cochrane Handbook, but not the original data.

## Conclusions

Our analysis showed that supplementation of ω-3 PUFA was efficacious in patients with NAFLD in decreasing ALT and TC, especially in decreasing TG, and increasing HDL-C. We also showed a tendency for ω-3 PUFA to decrease AST, GGT and LDL-C. However, ω-3 PUFA did not significantly improve liver fibrosis in NAFLD patients. More RCTs should be conducted to study the efficacy of ω-3 PUFA for stopping the progression of NAFLD and NASH.

## Supporting Information

S1 File PRISMA 2009 checklist. (DOC)

S2 File Search strategies in details. (DOC)
