# Supplementary material for: Effectiveness of Omega-3 Polyunsaturated Fatty Acids in Non-Alcoholic Fatty Liver Disease: A Meta-Analysis of Randomized Controlled Trials
Source: PLoS One. 2016 Oct 6;11(10):e0162368. doi: 10.1371/journal.pone.0162368 (PMC5053538; doi:10.1371/journal.pone.0162368)
Supplement: S2 File — (DOC) [file pone.0162368.s002.doc]

# S2_File Search strategies in details

# Medline Pubmed

(fish oil OR EPA OR eicosapentaenoic acid OR DHA OR docosahexaenoic acid OR omega-3 PUFA OR n-3 PUFA OR ω-3 PUFA) AND (NAFLD OR fatty liver OR hepatic steatosis OR steatohepatitis OR NASH) AND (randomized controlled trial OR controlled clinical trial OR randomized OR placebo OR clinical trials OR randomly OR trial)

Embase

('fish oil' OR 'EPA' OR 'eicosapentaenoic acid' OR 'DHA' OR 'docosahexaenoic acid' OR 'omega-3 PUFA' OR 'n-3 PUFA' OR 'ω-3 PUFA') AND ('NAFLD ' OR 'fatty liver' OR 'hepatic steatosis' OR 'steatohepatitis' OR 'NASH') AND ([controlled clinical trial]/lim OR [randomized controlled trial]/lim OR [meta analysis]/lim)

# Cochrane library

(fish oil OR EPA OR eicosapentaenoic acid OR DHA OR docosahexaenoic acid OR omega-3 PUFA OR n-3 PUFA OR ω-3 PUFA) AND (NAFLD OR fatty liver OR hepatic steatosis OR steatohepatitis OR NASH) AND (RCT OR randomi$ez controlled trial* OR clinical trial* OR meta analys$s)

**Refined by:** **RESEARCH DOMAINS:** (SCIENCE TECHNOLOGY) AND **RESEARCH AREAS:** ( GAATROENTEROLOGY OR PHARMACOLOGY PHARMACY OR NUTRITION )

**Timespan:** All years.

Search language=Auto
